# Modified minimally invasive extensor carpi radialis longus tenodesis for scapholunate dissociation: a prospective observational study

**DOI:** 10.1186/s12891-017-1414-7

**Published:** 2017-01-31

**Authors:** Alexander Kaltenborn, Sebastian Hoffmann, Andreas Settje, Peter M. Vogt, André Gutcke, Mike Rüttermann

**Affiliations:** 1 Department of Trauma and Orthopedic Surgery, Section for Reconstructive and Hand Surgery, Federal Armed Forces Hospital Westerstede, Lange Strasse38, Westerstede, 26655 Germany; 2 Core Facility Quality Management and Health Technology Assessment in Transplantation, Integrated Research and Treatment Centre Transplantation (IFB-Tx), Hannover Medical School, Hannover, Germany; 3 Institute of Hand and Plastic Surgery Oldenburg, Oldenburg, Germany; 4 Department of Plastic, Aesthetic, Hand and Reconstructive Surgery, Hannover Medical School, Carl-Neuberg-Str. 1, Hannover, 30625 Germany; 5 Department of Plastic Surgery, University Medical Centre Groningen, University of Groningen, Groningen, The Netherlands

**Keywords:** Scapholunate dissociation, ECRL-tenodesis, Minimal invasive technique, Wrist surgery, Carpal instability, Wrist trauma

## Abstract

**Background:**

Scapholunate dissociation is the most common form of carpal instability. However, there is no gold standard for operative treatment. In this prospective observational study on 54 patients, a modified minimally invasive dynamic extensor carpi radialis longus tenodesis is described, which is characterized by a smaller approach and application of a cannulated screw and washer for tendon fixation.

**Methods:**

Quick-Disabilities of Arm, Shoulder and Hand (DASH)-questionnaire results, post-operative satisfaction, range of motion and grip strength are analyzed.

**Results:**

A median Quick-DASH of 54.6 was observed pre-operatively which significantly improved to a median of 28.4 after the procedure (*p* < 0.001). Median follow-up was 24 months. Of 46 completely followed-up patients, 31 patients (67.4%) reported that they were satisfied with the outcome. Thirty-seven patients (80.4%) would recommend the procedure to a friend. Thirty-five patients (76.1%) reported some kind of complaint in the operated hand during follow-up. There was no association of severity of symptoms and co-morbidities with the outcome. Neither palmar flexion, nor dorsal extension was significantly different between the operated and non-operated wrist. The operated wrists were observed to have less grip strength than non-operated wrists.

**Conclusions:**

The presented method seems to be as successful as other techniques described in literature. It is less invasive, thus more patient friendly without harming feasibility of future salvage options. However, post-operative complaint rate was quite high.

## Introduction

Scapholunate dissociation (SLD) is the most common form of carpal instability. It is a result of a partial or complete rupture of the scapholunate ligament [1]. The frequency of concomitant SLD with distal radial fractures and/or scaphoid fractures is reported in the literature ranging from 7 to 69% [2–4]; when more complex trauma occurs such as lunate dislocation respectively perilunate dislocation, SLD is a frequently associated injury [4]. The diagnosis is often missed in seriously traumatized patients, especially due to the often unspecific symptomology and a missing primary radiological correlation. Untreated SLD leads to palmar flexion of the scaphoid and dorsal flexion of the lunate, which in the long run results in dorsal intercalated segment instability (DISI) and painful carpal arthrosis [5].

Several operative techniques have been reported for SLD management, the therapeutic method of choice depends on the stage of carpal instability, the status of surrounding joints and the patients’, as well as local preferences [6]. The different procedures ranging from arthroscopic repair methods, temporary fixations with either K-wires or screws, capsulodesis- and tenodesis-techniques to bone-tissue-bone repairs (BTB), to intercarpal fusion and proximal row carpectomies [1, 6–8]. However, reported results are ambiguous and the optimal method of choice is still a matter of controversy.

In this prospective-observational study, a modified, minimally invasive dynamic extensor carpi radialis longus (ECRL)-tenodesis for treatment of SLD is described, which is a modification based on a technique presented by Bleuler and colleagues in 2008 [9].

## Patients and methods

This prospective, observational, single-center study included 54 patients, who underwent operative treatment for symptomatic SLD with a modified, minimally invasive ECRL-tenodesis technique, which was based on the dynamic ECRL-tenodesis first described by Bleuler and colleagues [9].

The study was registered on www.researchregistry.com received the Research Registration Unique Identifying Number 988. The Westerstede clinical center institutional review board agreed on the study protocol, informed consent was obtained from each patient before inclusion.

### Inclusion and exclusion criteria

All consecutive operations of symptomatic SLD patients applying the new technique between September 2008 and June 2012 were considered for inclusion in this study. Only patients who did not improve under conservative treatment such as hand therapy, splinting or injections were treated operatively and included in this study. Thus, there were no patients included with acute wrist trauma. Excluded from the outcome analyses were 9 patients (16.4%), who were not available for post-operative examination and questioning and thus were lost to follow-up. These patients did not answer our inviation letters and calls to visit the outpatient clinic. Written informed consent was obtained from all patients prior to inclusion.

Scapholunate dissociation was categorized in three stages according to recent guidelines of the German Society for Hand Surgery, SLD I° - pre-dynamic stage, SLD II° - dynamic stage, and SLD III° - static stage [10]. Mean follow-up was 24 months after surgery (median 24; range 2.5–46.5 months).

### Modified Bleuler technique of ECRL-tenodesis

**Fig. 1:**
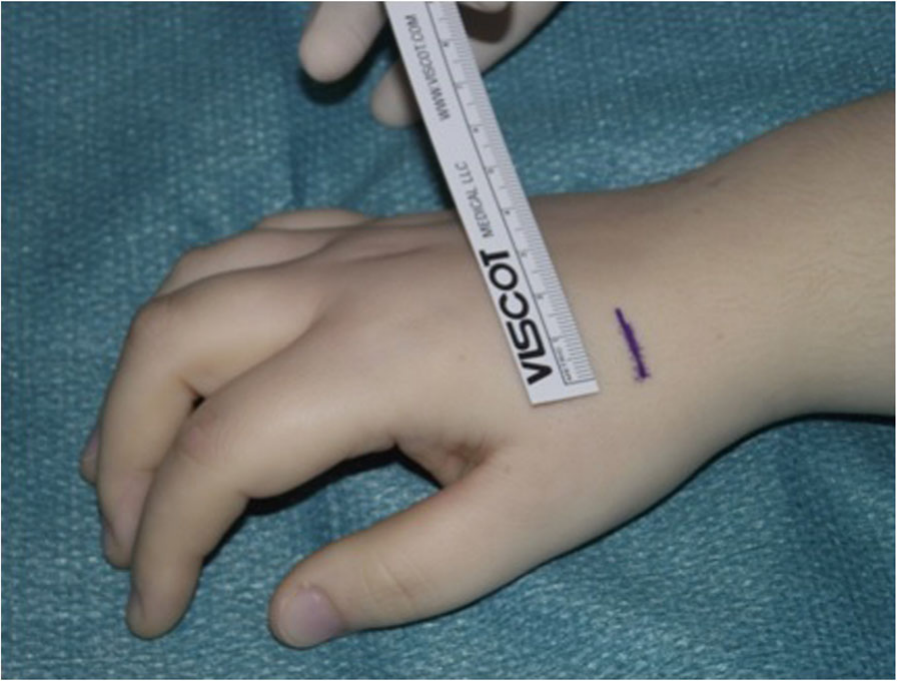
Surgical approach chosen for the presented minimally invasive ECRL-tenodesis

**Fig. 2:**
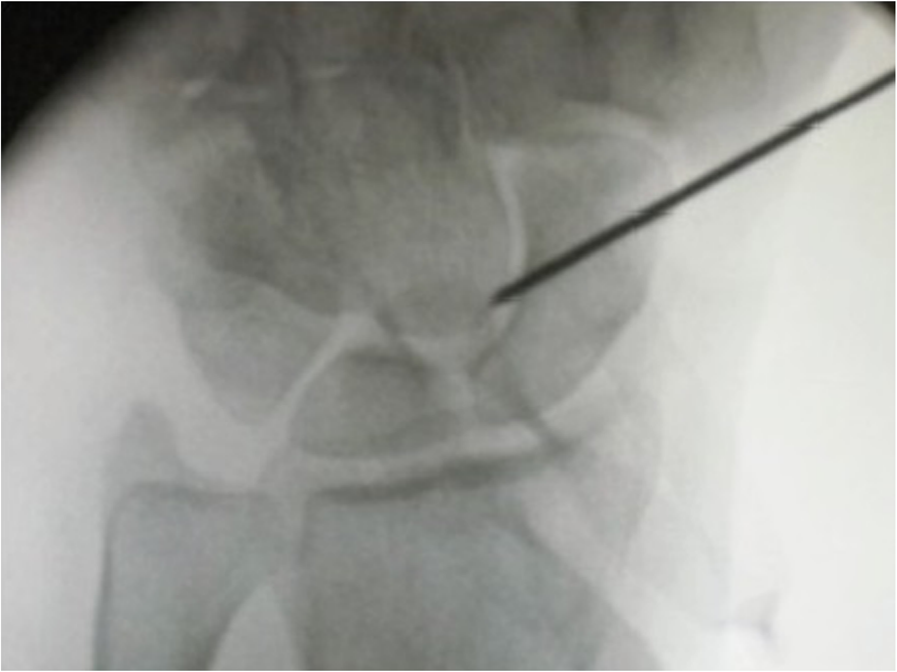
Shown is the K-wire insertion into the distal scaphoid

**Fig. 3:**
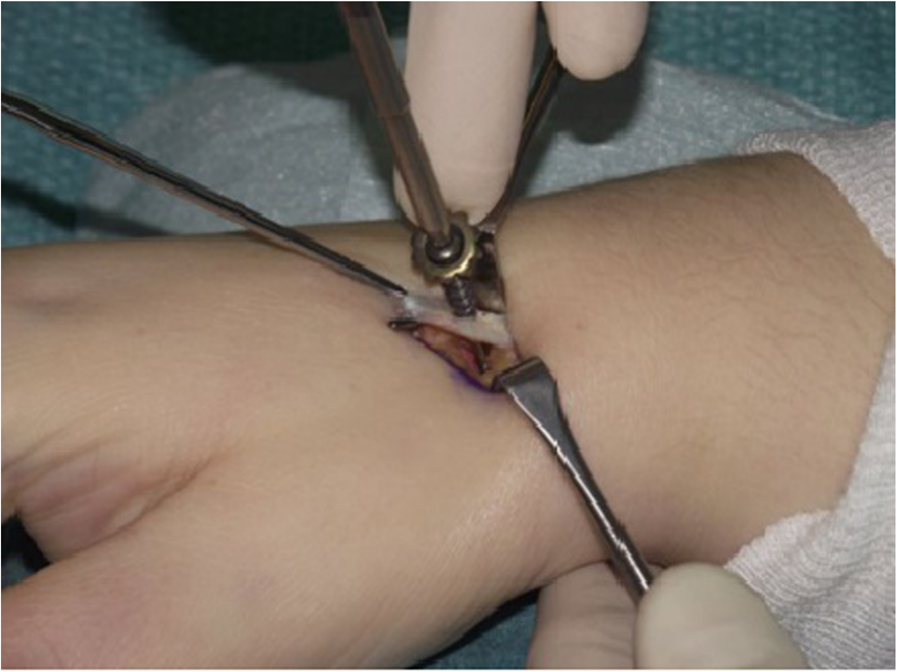
A cannulated screw and spike-edged titan washer is applied for fixation of the ECRL tendon

**Fig. 4:**
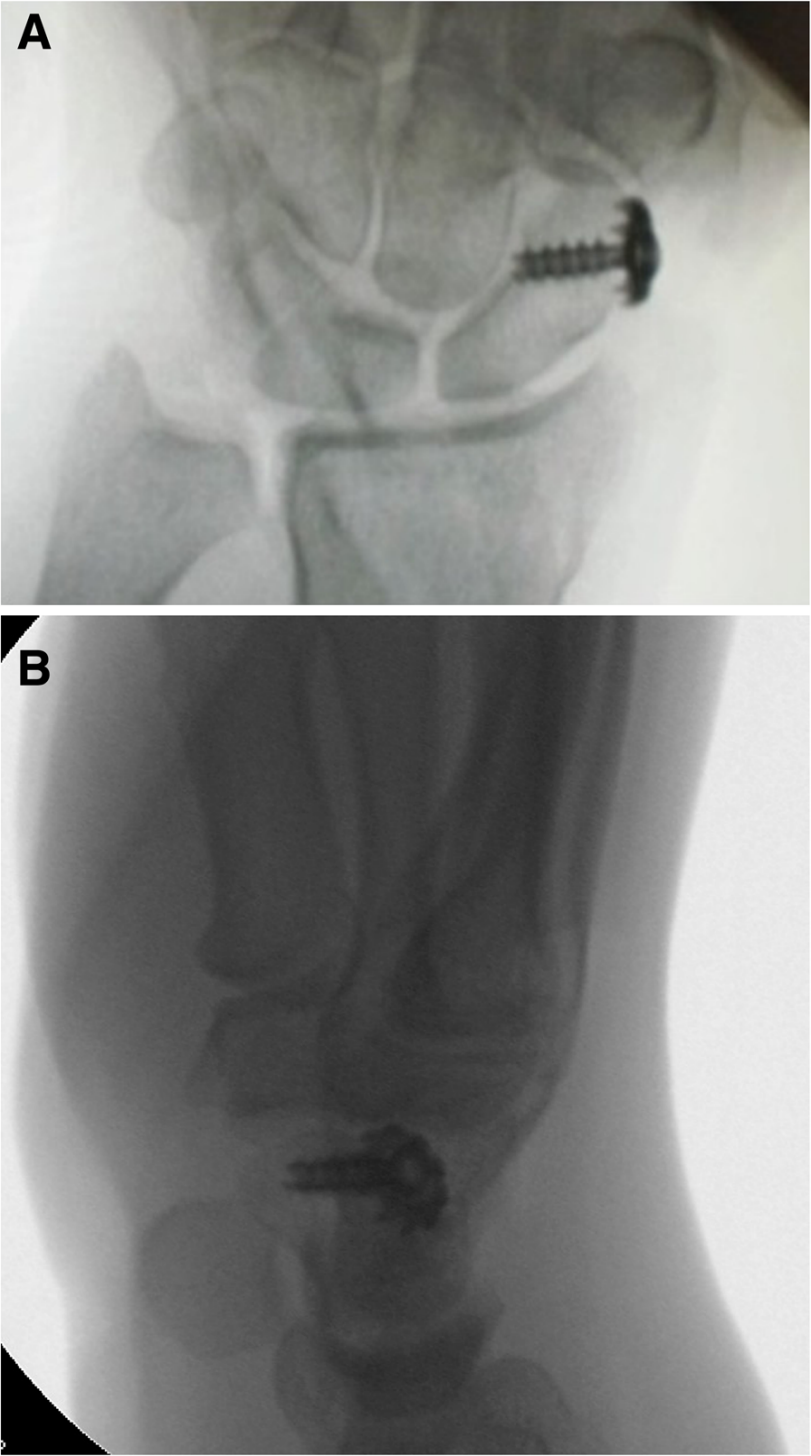
The result of ECRL-tendon fixation is checked with the image intensifier in a.p. (**a**) and lateral view (**b**)

In our institution, the operation is typically performed with plexus anesthesia and a bloodless field. In contrast to the technique by Bleuler and colleagues, a minimally invasive approach is applied in the presented method. Thus, a 2 cm incision is made on the dorsoradial aspect of the wrist, approximately 1 cm distal to the radial styloid (see Fig. 1). Sparing the superficial branch of the radial nerve, the second extensor compartment is dissected and incised to mobilize the ECRL-tendon. The dorsal capsule is opened at the STT-joint and a 0.8 mm K-wire is inserted into the distal scaphoid. This is pointed in direction of the scaphoid tubercle which is checked with the image intensifier (see Fig. 2). Over the K-wire a cannulated 2.7 mm drill is then inserted. After drilling, a small piece of the cortex around the drilling hole is removed with a rongeur, which facilitates tendon insertion and adhesion to the bone. The longitudinally split ECRL-tendon is now pulled in distal direction over the drill hole and fixated at the distal scaphoid with a 4 mm cannulated ASNIS titanium screw (Stryker, Kalamazoo, Michigan, USA) and a titan mini-washer with spiked edges through the tendon incision (Fig. 3 and 4a/b), while the scaphoid is repositioned with manual pressure from the palmar side. This is done as follows: In neutral position of the wrist the ECRL tendon is pulled as far as possible with a tendon retractor while the scaphoid is repositioned through a Watson maneuver. Then the screw and the washer are placed over the K-wire until the tendon is firmly pressed to the scaphoid bone.

**Fig. 5:**
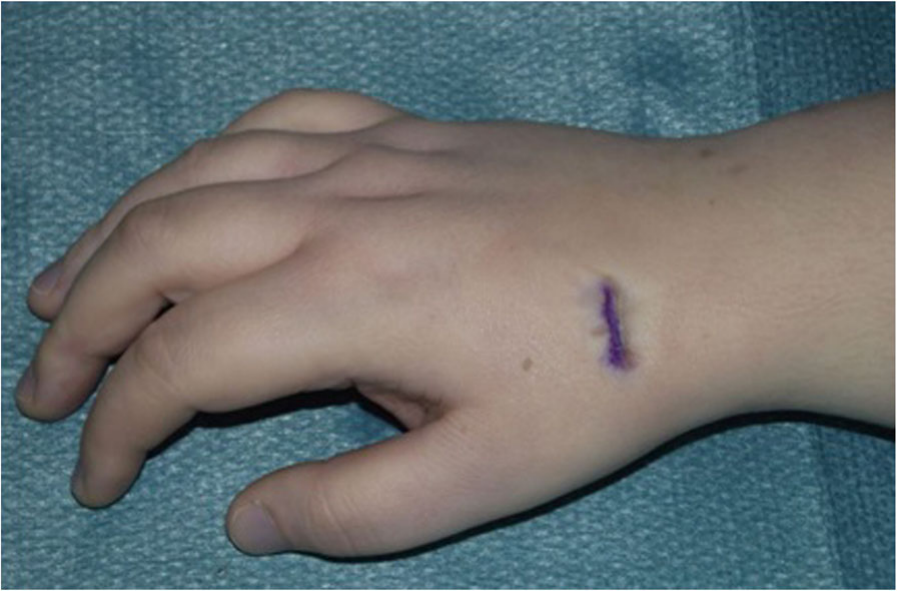
Shown is the closed skin incision after modified minimally invasive ECRL-tenodesis

The length of the screw is normally 14–16 mm and should be chosen so that the screw head has good contact to the cortex. The result and especially the reduction of DISI deformity is checked with the image intensifier. After suture of the capsule and the incision, a dorsal forearm splint is applied in 30° dorsal extension in the wrist (Fig. 5). Handtherapy started 6 weeks postoperatively, and forced palmar flexion of more than 40° should be avoided for further 3 weeks.

In summary, the modification of the original technique by Bleuler and colleagues comprises a reduced, minimally invasive approach and the application of a cannulated screw and washer with spiked edges for secure tendon fixation. Both, the small approach as well as the application of such washer, are alterations to the original technique. With these aspects it is intended to reduce the rate of post-operative tendon dislocations and a more stable SL-reduction. The joint was not approached during the procedure, e.g. for diagnosis confirmation, to ensure the benefits of minimal invasiveness. The SLD was diagnosed clinically with a positive Watson test pre-operatively and under local anesthesia, radiological evaluation and wrist arthroscopy, which was performed in all included patients before minimally invasive ECRL-tenodesis.

### Study endpoints

Primary study endpoint was hand function as measured via range of motion and grip strength assessed with a hydraulic hand dynamometer (SH5001, Saehan Cooperation, Changwon, South Korea), as well as symptoms after modified ECRL-tenodesis measured with Quick-Disabilities of the Arm, Shoulder and Hand (Quick-DASH) Questionnaire including the high performance Sport/Music as well as Work sections [11, 12]. Additionally, patients were asked to categorize their post-operative satisfaction in one of 5 stages: one - fully satisfied, two - satisfied, three - indifferent, four - unsatisfied, five - fully unsatisfied; as well as whether they would repeat the treatment or recommend it to a friend or not. Secondary study endpoint was the onset of post-operative complications.

### Statistical analysis

Consecutive data were presented as mean and standard deviation (SD) if normally distributed and median and range if not normally distributed. Normal distribution was assessed by application of the Kolmogorov-Smirnov test. Normally distributed data were compared with paired two-sided t-tests. The Mann–Whitney-U test was applied for comparison of non-parametric data. Correlation between patient satisfaction and patient characteristics was analyzed using Pearson’s Chi^2^-tests where applicable. For the identification of risk factors for the onset of post-operative complaints, univariate binary logistic regression was applied. To control for possible confounding factors with influence on outcome parameter, a multivariable principal component analysis was applied. In this graphical analysis, the effects of evaluated variables on the study endpoint are depicted as diverging vectors, which imply statistical independence in the multivariable model. For all statistical tests a *p*-value <0.05 was defined as significant. The SPSS statistics software 23.0 (IBM, Somers, NY, USA) was used to perform statistical analyses.

## Results

Descriptive statistics of the investigated study population are summarized in Table 1. A median Q-DASH of 54.6 (range: 9–86) was reported as measurement for physical function pre-operatively. After operative treatment with the modified ECRL-tenodesis, the Q-DASH score significantly improved to a median of 28.4 (range: 0–89) (*p* < 0.001) after a mean of 24 months of follow-up. A significant improvement could also be shown for the Q-Dash work module from a median of 62.5 (range: 0–100) pre-operatively to 25.0 (range: 0–75) post-operatively (*p* < 0.001). The median Q-DASH sports/music module-score significantly improved from 71.9 (range: 19–100) pre-operatively to a median of 25.0 (range: 0–100) post-operatively (*p* = 0.001). A mean follow-up of 24 months was observed (SD: 12.4 months).

**Table 1:** Descriptive statistics of the study population (*n* = 54) at the time of operative treatment

	Median [range]	*n*	% of cohort
Male		36	67
Female		18	33
SLD I°		1	2
SLD II°		11	20
SLD III°		43	78
History of distal radius fracture		6	11
Age in years	28.5 [17.7–63.9]		

Table 2 summarizes the results of post-operatively achieved range of motion in the wrist in comparison to the not operated opposing wrist. There was a significantly decreased grip strength and ulnar deviation observed (both *p* < 0.001).

**Table 2:** Summary of the results of postoperative range of motion as well as grip strength of the operated side in comparison to the opposing wrist

Motion	Operated hand mean (SD)	Opposing side mean (SD)	*p*-value^a^
Dorsal extension	55.5 (16.8)	68.3 (10.4)	0.072
Palmar flexion	65.5 (18.3)	72.9 (13.5)	0.054
Radial deviation	13.0 (7.2)	25.1 (7.5)	0.327
Ulnar deviation	35.8 (12.2)	45.3 (9.7)	<0.001
Grip strength in kg	30.2 (14.9)	44.3 (13.9)	<0.001

^a^Paired two-sided t-test

In binary regression analysis, pre-operative Q-DASH results have no significant association to post-operative outcome.

Of 46 followed-up patients, 31 patients (67.4%) reported that they were fully satisfied or satisfied with the outcome of the surgical treatment. 7 patients (15.2%) reported to be indifferent to the operations’ results. Overall, 8 patients (17.4%) reported to be unsatisfied (*n* = 7, 15.2%) or fully unsatisfied (*n* = 1, 2.2%) after operative treatment with modified ECRL-tenodesis. This results in a median satisfaction level of 2.0 (satisfied) (range: 1–5).

Thirty-five patients (76.1%) reported that they would repeat the operative treatment if suffering from the same symptoms again. Moreover, 37 patients (80.4%) would recommend the modified ECRL-tenodesis to a friend or family member as treatment for SLD.

The investigated patients received post-operative treatment (e.g. handtherapy) for a median of 3.5 months (range: 1–15 months), which usually started after 6 weeks of cast immobilization. Patients reported to be back at work after a median of 3 months (range: 1–16 months). All patients returned to their previously conducted work.

### Observed post-operative complaints and risk factors

Thirty-five patients (76.1%) reported of some kind of complaint in the operated hand during follow-up. The most common phenomenon that patients reported was screw-impingement, which was described as painless blocking sensation in the area of the operated tissue during radial deviation and/or hyperextension of the wrist and was optionally treated with removal of the screw. Screw-impingement occurred in 20 patients (43.5%). 5 patients (10.9%) reported temporary dysesthesia in the area of the operative approach, potentially affecting the radial superficial nerve, that did not need treatment. In two cases (4.3%), a partially ruptured ECRL-tendon could be revealed as cause for wrist pain, which was re-fixated in a re-operation with good results regarding patient satisfaction (*p* = 0.042). Development of a neuroma, screw failure, as well as complex regional pain syndrome were reported in one patient each (2.2%). None of the patients reported a recurrence of symptoms. There was no case of avascular necrosis of the scaphoid during follow-up.

**Fig. 6:**
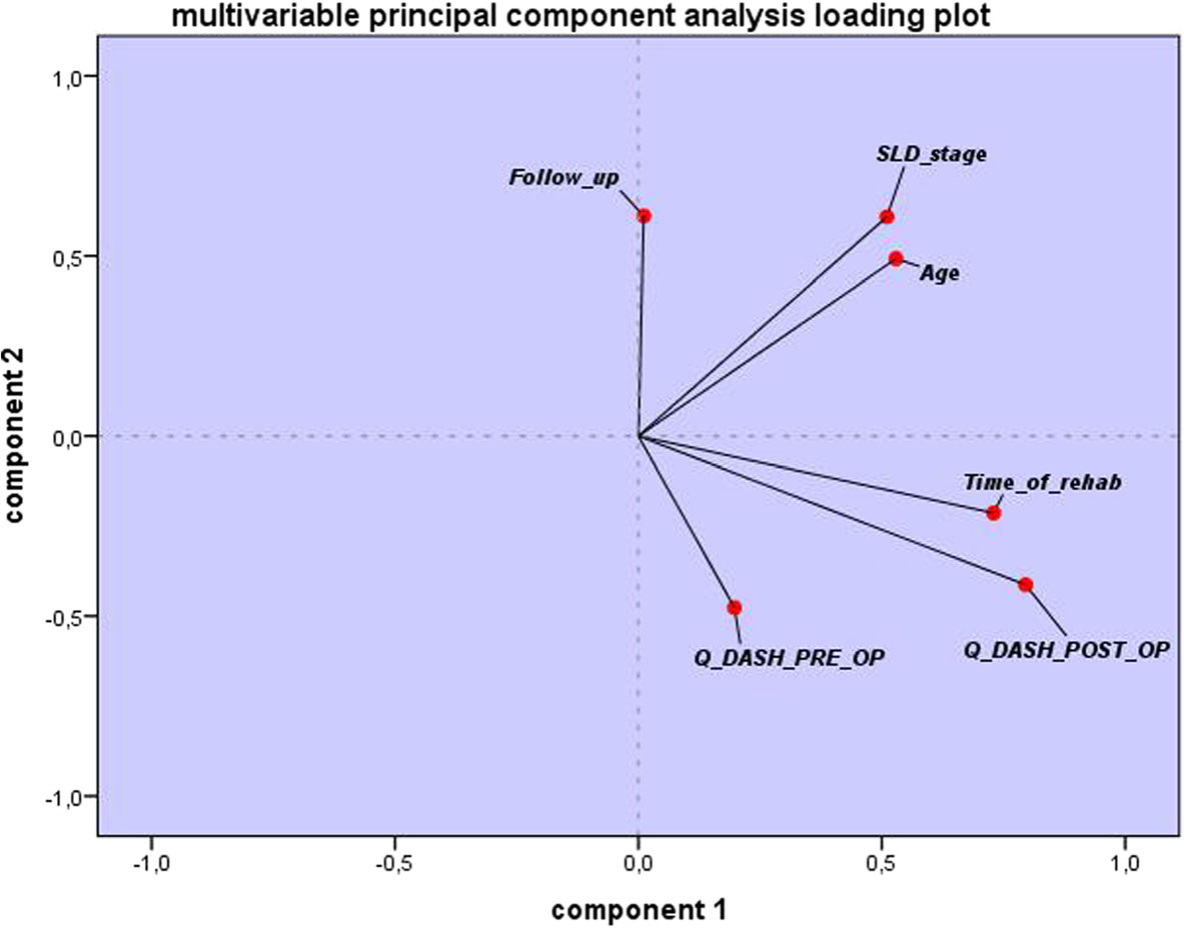
Multivaribale principal component analysis shows the independence of improved outcome of possible confounders. In this graphical analysis, the effects of evaluated variables on the study endpoint are depicted as diverging vectors, which imply statistical independence in the multivariable model

Multivariable principal component analysis revealed that the improved outcome regarding the Q-DASH is independent from possible confounders, specifically pre-operative severity of symptoms, SLD-stage, length of follow-up, patient age as well as length of post-operative rehabilitation (see Fig. 6).

## Discussion

Multiple procedures and techniques have been described for the operative treatment of SLD. Their application mostly depends on the SLD-stage and reported results are ambiguous and variable [1–10]. Therefore, the optimal method of choice remains a matter of controversy and there is still no real gold standard treatment for SLD. This is especially true for patients suffering from SLD irresponsive to conservative treatment and higher stages of dissociation, which is exactly the examined patient group, who had a mean scapholunate diastasis of 3.5 mm (SD: 1.2) pre-operatively, representing a fully torn ligament according to Lindau’s classification [13].

The presented method is quick and can be done under regional anesthesia in day care. There is no need for K-wire stabilization and thus any secondary procedure or associated complications. Furthermore, it is minimally invasive and does not impede other more invasive options, as three ligament tenodesis [14] or reduction and association of the scaphoid and lunate (RASL)-method [15]. More invasive options like midcarpal/four-corner fusion or proximal row carpectomy will also not be influenced at all by this procedure which makes it a valuable choice in symptomatic patients.

The proportion of patients suffering from static SLD in this study is rather high (78%), Ross and colleagues investigated 72% static SLD and Garcia-Elias reported a 21% proportion in their cohorts regarding operative treatment options for SLD [14, 16]. Strikingly, this is not resulting in poorer outcome in the presented study despite these more challenging patient characteristics. It needs to be taken into account that the aim of operative treatment in the current cohort was reduction of wrist pain and to regain of functionality. This is represented in the improved Q-DASH results and a return to work rate of 100%.

One of the main criticisms of any procedure for SLD which relies on a dorsal check rein is the limitation of palmar flexion in the wrist. Nevertheless, the presented method did not lead to a significantly impaired range of motion in wrist flexion and extension, although there is a trend towards slightly reduced range of motion without reaching statistical significance. Moreover, it needs to be kept in mind that grip strength as well as ulnar deviation are significantly reduced in comparison to the not affected wrist, which should be reported to the patients pre-operatively to allow a well-informed consent (see Table 2).

The dynamic ECRL-tenodesis enhances the extension forces on the scaphoid in all wrist positions thus allowing more benefit in terms of the wrist throughout the entire range of motion. Furthermore, it makes a partial wrist fusion redundant, which seems to prevent early occurrence of arthrosis in adjacent joints [9]. The investigated patient group was followed up for an average of 2 years with a maximum of almost 4 years in the current study. Thus, the presented data suggests that the excellent preliminary results reported by Bleuler et al. seem to remain during longer follow-up. In comparison with the original Bleuler-technique, the modifications are a reduced, minimally invasive approach and the application of a cannulated screw and washer with spiked edges for better tendon fixation.

This leads to a majority of satisfied patients, which were all able to return to work after a reasonable timeframe. These overall results regarding the outcome are comparable with Bleuler’s observations from 2008 [9]. The current technique further allows achievement of sufficient stability in the wrist as well as only minimal postoperative reduction of wrist movement. However, the post-operative complaint rate was quite high at 76.1%.

Interestingly, all investigated pre-operative parameters were not significantly associated to post-operative Q-DASH results, neither were pre-operative Q-DASH results (see Table 3).

**Table 3:** Summary of the results of univariate binary logistic regression analyses of pre-operative variables as possible risk factors for high post-operative Q-DASH results as well as for the onset of post-operative complaints

Pre-operative Variable	*p*-value; endpoint post-operative Q-DASH	*p*-value; endpoint post-operative complaints
Patient gender	0.221	0.745
Affected hand	0.771	0.352
Stage of SLD	0.757	0.171
Age at operation	0.250	0.562
Time between operation and follow-up examination	0.439	0.381
Lunotriquetral instability	0.064	0.999
Radiocarpal osteoarthritis	0.155	0.683
History of distal radial fracture	0.637	0.462
Carpal tunnel syndrome	0.081	0.288
Mediocarpal osteoarthritis	0.999	0.322
Tenosynovitis	0.999	0.446
Thumb basal joint osteoarthritis	0.999	0.683
Scapholunate advanced collapse wrist	0.149	0.276
Lunate dislocation	0.312	0.446
Cubital tunnel syndrome	0.312	0.446
Dupuytren’s disease	0.312	0.466
Scaphotrapezotrapezoidal osteoarthritis	0.312	0.182
Q-DASH	0.955	0.403
Q-DASH work	0.931	0.426
Q-DASH sport	0.879	0.399

The fact that patients’ satisfaction was also not significantly correlated to age (*p* = 0.813), pre-operative Q-DASH score (*p* = 0.782), gender (*p* = 0.588), stage of SLD (*p* = 0.566), or one of the observed concomitant morbidities shows that the presented method might well have a good prospect for a diverse group of patients. To investigate whether possible confounders might influence the analysis, a multivariable principal component analysis was applied to control statistically for these parameters. It could be shown that there might be a slight co-linearity between the SLD-stage and patient age. However, none of the analyzed variables, e.g. length of follow-up or patient age showed a confounding effect on the outcome (see Fig. 6). It can be concluded that the improvements shown in this study are independent effects of the applied ECRL-tenodesis and are independent from these possible confounding factors.

The relatively high post-operative complaints rate is most probably due the high level of surveillance which was defined by our prospective study protocol. It needs to be noted, that most of the reported complaints were due to subjective screw-impingement, which was painless in all cases, and temporary dysesthesia in the operated region, which disappeared during follow-up without further treatment.

Moreover, the reported complaints did not lead to a significantly impaired overall outcome in terms of functionality measured with the Q-DASH (*p* = 0.242) or patient satisfaction (*p* = 0.632). This assumption is supported by the fact that 76% of patients would repeat the procedure and more than 80% of patients would recommend it to a friend or family member as treatment for SLD. Hence, it can be concluded that the applied operative technique is overall successful regarding the study endpoints, especially since none of the investigated patients reported on the recurrence of pre-operative symptoms.

A major focus of the presented study was qualitative outcome regarding patient satisfaction and their ability to return to work post-operatively as well as functional outcome measured via Q-DASH. The observed overall satisfaction of 67% of followed-up patients is comparable to the results of e.g. Garcia-Elias and colleagues, who reported a recovery of a painless functional wrist in 70% of their investigated cohort [14], although their technique is more invasive.

The recently published data by Ross et al. did not report on qualitative outcome regarding patient satisfaction, hence a comparison with current results is not yet possible [16]. However, they presented good results of regained grip strength and Q-DASH post-operatively.

The RASL-procedure as described by Rosenwasser and co-workers as early as 1997 is assumed to be ineffective in providing sufficient stability about the SL-interval, as shown by a recently published study from Larson and Stern [15, 17]. Techniques basing on bone-to-bone grafting were evaluated by Harvey and colleagues [7]. Their latest data show a return rate to the pre-injury workplace of 80%, which is comparable with a return rate of 77% as most recently reported by Ho et al. for an arthroscopic-assisted combined dorsal and volar SL-ligament reconstruction [8]. In contrast to these result, 100% of investigated patients in the current study returned to their work place at their pre-injury job level.

Links and co-workers reported comparable results for functional outcomes in Q-DASH reduction, especially for their modified Brunelli-technique [18]. However, they did not provide data on post-operative patient satisfaction. Regarding post-operative range of motion and grip strength, the current results are comparable or favorable in comparison to other reported techniques [1]. For example, the three-ligament tenodesis presented by Garcia-Elias and colleagues in 2006 showed a good rate of pain reduction, although grip strength was reduced by one third of the uninjured wrist and the flexion and extension was reduced by nearly 50% [14]. Moran et al. analyzed grip strength and range of motion after intercarpal capsulodesis and flexor carpi radialis tenodesis using a modified Brunelli technique in the operated wrist in comparison with the unaffected side and reported results which are comparable to the current study’s technique [19].

The current study also has limitations. The patient cohort is limited, as is the follow-up time. It is intended to follow-up the investigated study cohort for long-term results. Moreover, further studies with more patients will be needed to validate the presented results and compare these with other methods, e.g. B-T-B repairs or the modified Brunelli-reconstruction, as well as minimally invasive arthroscopic techniques [7, 8, 18, 20]. The grip strength as well as ulnar deviation were shown to be significantly reduced in the operated wrist when compared to the healthy opposing hand (both *p* < 0.001). It needs to be evaluated, whether these observed limitations can be confirmed after longer follow-up. Unfortunately, data on pre-operative range of motion was not available at the time of analyses. Future studies should be outlined focusing on the reduction of post-operative complaint rate and should also include further methods such as intention-to-treat analysis to confirm the promising results of the current data.

## Conclusions

In conclusion, the presented results show that the modified minimally invasive dynamic ECRL-tenodesis is a feasible and successful method for the treatment of SLD in patients which are non-responsive to conservative treatment regardless of their age, SLD stage or pre-operative severity of symptoms and functionality. Results are comparable to more invasive methods that have more surgical impact on the patient and leave less options for treatment of future aggravation of (mid) carpal osteoarthritis than the presented method. Therefore, the presented operative technique can be recommended, especially for patients with higher stages of symptomatic SLD. However, post-operative complaint rate was quite high.

## Abbreviations

BTB: Bone-to-bone
DASH: Disabilities of the arm, shoulder and hand
DISI: Dorsal intercalated segment instability
ECRL: Extensor carpi radialis longus
RASL: Reduction and association of the scaphoid and lunate
SD: Standard deviation
SLD: Scapholunate dissociation
